# Work-Related Noise Exposure in a Cohort of Patients with Chronic Tinnitus: Analysis of Demographic and Audiological Characteristics

**DOI:** 10.3390/ijerph14091035

**Published:** 2017-09-08

**Authors:** Massimo Ralli, Maria Paola Balla, Antonio Greco, Giancarlo Altissimi, Pasquale Ricci, Rosaria Turchetta, Armando de Virgilio, Marco de Vincentiis, Serafino Ricci, Giancarlo Cianfrone

**Affiliations:** 1 Department of Oral and Maxillofacial Sciences, Sapienza University of Rome, Viale del Policlinico 155, 00186 Rome, Italy; 2 Department of Anatomy, Histology, Legal Medicine and Orthopaedics, Sapienza University of Rome, Viale Regina Elena 336, 00161 Rome, Italy; mapab@libero.it (M.P.B.); pasquale.ricci@uniroma1.it (P.R.); serafino.ricci@uniroma1.it (S.R.); 3 Department of Sense Organs, Sapienza University of Rome, Viale del Policlinico 155, 00186 Rome, Italy; antonio.greco@uniroma1.it (A.G.); giancarlo.altissimi@uniroma1.it (G.A.); rosaria.turchetta@uniroma1.it (R.T.); armando.devirgilio@uniroma1.it (A.d.V.); marco.devincentiis@uniroma1.it (M.d.V.); giancarlo.cianfrone@uniroma1.it (G.C.)

**Keywords:** noise-induced hearing loss, tinnitus, occupational noise exposure, pure tone audiometry

## Abstract

Work-related noise exposure is one of the major factors contributing to the development of adult-onset hearing loss and tinnitus. The aim of this study was to analyze, in patients with chronic tinnitus and long-term occupational noise exposure, (A) characteristics of hearing loss, tinnitus, comorbidities, demographic characteristics and a history of work-related noise exposure and (B) differences among individuals employed in occupations with high and low risk of developing work-related noise-induced hearing loss (NIHL). One hundred thirty six patients with chronic tinnitus and at least a 10 year-long working history were divided into two groups based on the risk of their profession to induce NIHL. Individuals employed in jobs at high risk for NIHL were mostly males and exhibited a poorer hearing threshold, more evident in the left ear. Tinnitus was mostly bilateral; the next largest presentation was left-sided; patients described their tinnitus as buzzing or high-pitched. Correlation between age, length of tinnitus and worse hearing was found. Patients with a higher degree of hearing impairment were mostly males and were more likely to have a family history of hearing loss and at least one cardiovascular comorbidity. Our study shows some differences in individuals with tinnitus and a history of a profession associated with increased exposure to NIHL compared to those without such a history.

## 1. Introduction

Noise-induced hearing loss (NIHL), commonly defined as a hearing threshold worse than 25 dB HL at the high-frequency range [1], is a major cause of hearing impairment. Workplace noise exposure is an important risk factor of NIHL in workers; 16% of disabling adult-onset hearing loss worldwide is attributed to occupational noise [2,3]. NIHL is the most frequent work-related disorder in the United States [4,5]. 

Chronic exposure to loud noise induces a progressive destruction of inner and outer hair cells in the organ of Corti and alterations to the stria vascularis and spiral ganglion neurons. The mechanism of noise-induced hearing loss begins with outer and, to a lesser extent, inner hair cell loss in the high-frequency base of the cochlea, followed by a progression of hair cell loss toward the low-frequency apex of the cochlea [6,7,8]. Oxidative stress, metabolic exhaustion, ischemia and ionic imbalance in the inner ear fluids play a central role in the pathophysiology of NIHL. Reactive oxygen species and reactive nitrogen species participate in cellular mechanisms that underlie hair cell death after noise exposure and lead to sensorineural hearing loss [9,10,11,12,13,14].

Tinnitus is defined as the perception of sound without an external auditory stimulus. Approximately 2% of the population in industrialized countries is reported to experience incessant tinnitus [15]. Tinnitus may have audiological, somatic or psychological bases [16,17,18,19,20,21,22,23,24]; risk factors for tinnitus include hearing loss, exposure to loud noise and increasing age [25,26,27]. Furthermore, patients often report worsening of tinnitus with stress; therefore, workers subject to high job stress may have an increased risk of tinnitus [28,29,30]. Hearing loss is the most common cause of tinnitus; in patients with NIHL, rates of tinnitus range from 35 to 77% [31,32]. Occupational noise has a role in contributing to the development of tinnitus [33]. 

The effects of long-term occupational noise in patients suffering from chronic tinnitus have rarely been studied, and limited information is available for specific occupation groups [34]. The aim of this study was to analyze in a cohort of individuals with chronic tinnitus (A) the characteristics of hearing loss, tinnitus, comorbidities, demographic variables and a history of work-related noise exposure and (B) differences among individuals employed in occupations with high and low risk of developing work-related NIHL.

## 2. Materials and Methods

In this study, we included 136 patients aged 26–84 years with chronic tinnitus (>12 months) and anamnestic history of having worked at least 10 years during the previous 20 years, presenting at the Tinnitus Unit of the Sapienza State University Hospital Policlinico Umberto I in Rome, Italy, during a 4-year period from January 2013–January 2017. 

Based on working history, patients were divided into two groups: patients with tinnitus and a history of employment in one of the professions associated with an increased exposure to occupationally-acquired noise-induced hearing loss (HIGH-RISK, *n* = 68) and patients with tinnitus and a history of employment in industries and occupations reported to have lower risks for hearing impairment (LOW-RISK, *n* = 68). Patients were included in the HIGH-RISK group if they had a history of employment in one of the following professions: armed forces [35,36,37,38,39,40,41,42], carpenters [36,38,43], manufacturing workers [5,34,35,43,44,45,46], drivers [5,34,38,43,47,48], miners [5,35,38,43,49,50], musicians [38,51,52,53], railroaders [4,5,34,43,54,55], school teachers [5,34,43] and construction workers [5,34,38,43,55,56,57,58]. Patients were included in the LOW-RISK group if they had a history of employment in one of the following occupations: entrepreneurs, hospital workers, office workers, professionals [4,5,29,59,60]. Exclusion criteria were a history of prolonged treatment with ototoxic drugs, middle or inner-ear disease (e.g., otosclerosis, chronic suppurative otitis media or endolymphatic hydrops), retrocochlear disease (e.g., vestibular schwannoma), previous ear surgery and psychiatric comorbidities.

Informed consent was obtained from each individual participant in the study. The study was conducted in accordance with the Declaration of Helsinki, and the protocol was approved by the Ethics Committee of the Sapienza University, Policlinico Umberto I, Rome. Patients underwent anamnestic interview and hearing evaluation through otoscopy, pure tone audiometry (PTA) and the acoustic immittance (AI) test. PTA was measured at frequencies of 0.50, 1, 2, 4 and 8 kHz.

Detailed work and noise-exposure history data were collected including type of work and family history for hearing loss and tinnitus. The presence of cardiovascular comorbidities such as diabetes, heart disease and hypertension was investigated.

Self-assessment questionnaires regarding tinnitus (Tinnitus Handicap Inventory (THI)) [61], hearing loss (Hearing Handicap Inventory (HHI)) [62] and hyperacusis (Hyperacusis Questionnaire (HQ)) [63,64] were administered during the initial visit. Tinnitus characteristics including side (unilateral, bilateral) and pitch from a predefined set of possibilities including “buzzing”, “whistle”, “high-pitched”, “low-pitched” and “other” were collected for each patient.

### Statistics

The mean and standard deviation (SD) for numeric and frequency and percentage for categorical demographic characteristics, such as sex, age, family history of hearing loss and comorbidities, distribution of tinnitus characteristics and self-administered questionnaire results, and PTA differences between high-risk and low-risk subjects were calculated. The chi-square test of association was used to analyze differences between the LOW-RISK and HIGH-RISK groups for demographic variables (age, sex) and tinnitus characteristics; *p*-values were reported. A multivariate binary logistic regression analysis was performed to investigate specific variables associated with a higher degree of hearing loss in tinnitus patients according to demographic characteristics such as age and sex, comorbidities, family history for hearing loss and self-administered questionnaire scores. The results of logistic regression were reported in the odds ratio scale along with a 95% confidence interval and *p*-values. A *p*-value of 0.05 was used as the cutoff for statistical significance.

## 3. Results

### 3.1. Demographics, Family History and Comorbidities

The study included 136 patients: 86 males (63.2%) and 50 females (36.7%). Males were significantly more prevalent in the HIGH-RISK group (55/68, 80.88% *p* < 0.001). In the LOW-RISK group, 31/68 were males (45.59%) and 37/68 were females (54.41%) (*p* < 0.001). 

Mean age was 55.1 years (range 26–84 years). Individuals in the HIGH-RISK group were older (56.6 years, range 31–81 years, SD = 12.4) compared to individuals in the LOW-RISK group (53.5 years, range 26–84 years, SD = 13.5) (*p* = 0.08). 

Mean time of noise exposure was 18.4 years in the LOW-RISK group and 19.3 years in the HIGH-RISK group. No statistically-significant difference was found between groups (*p* = 0.72).

Family history for hearing loss was found in 14/68 (20.6%) individuals in the HIGH-RISK group and in 9/68 (13.2%) in the LOW-RISK group; the difference was not statistically significant (*p* = 0.253). 

At least one comorbidity among diabetes, heart and vascular diseases and hypertension was found in 27/68 (39.7%) patients in the HIGH-RISK group and in 24/68 (35.3%) in the LOW-RISK group (*p* = 0.60); several patients presented more than one comorbidity. The most common comorbidity was hypertension, followed by heart and vascular diseases. Data are shown in Table 1. 

### 3.2. Hearing Loss

**Figure 1:**
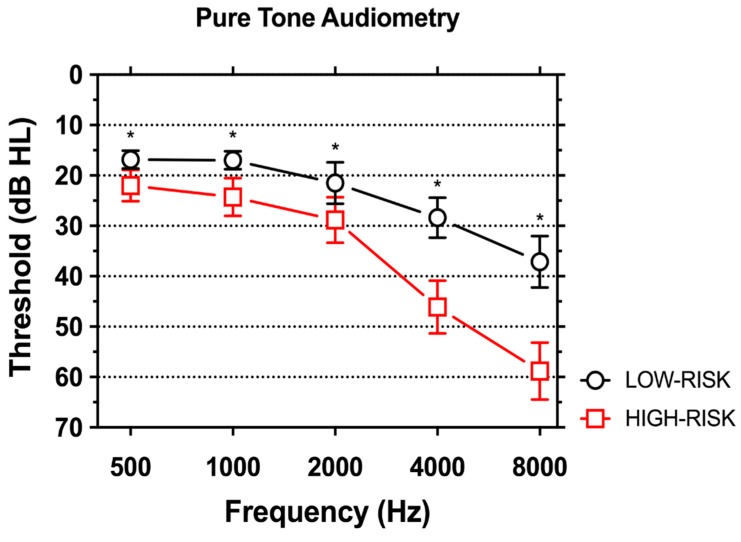
Pure tone audiometry in the LOW-RISK and HIGH-RISK groups. Means ±95 CI are shown. A statistically-significant worse auditory threshold was found for individuals in the HIGH-RISK group. Asterisks indicate statistically-significant differences. HL, hearing loss.

Figure 1 shows PTA in subjects with high and low risk of work-related NIHL. As expected, hearing was significantly worse in individuals in the HIGH-RISK group, especially for the frequencies between 2000 and 8000 Hz. 

Frequency-specific hearing thresholds are shown in Table 2. In the HIGH-RISK group, mean PTA thresholds were 22 dB HL for 500 Hz, 24.3 for 1000 Hz, 28.8 for 2000 Hz, 46.1 for 4000 Hz and 58.8 dB HL for 8000 Hz. In the LOW-RISK group, thresholds were 16.8 dB HL for 500 Hz, 17.0 for 1000 Hz, 21.5 for 2000 Hz, 28.4 for 4000 Hz and 37.1 dB HL for 8000 Hz. Mean PTA thresholds in the HIGH-RISK exceeded thresholds in the LOW-RISK group by 5.2 dB HL for 500 Hz, 7.3 dB for 1000 Hz, 7.3 dB for 2000 Hz, 17.7 dB for 4000 Hz and 21.7 dB for 8000 Hz. Differences were statistically significant for each frequency. 

**Figure 2:**
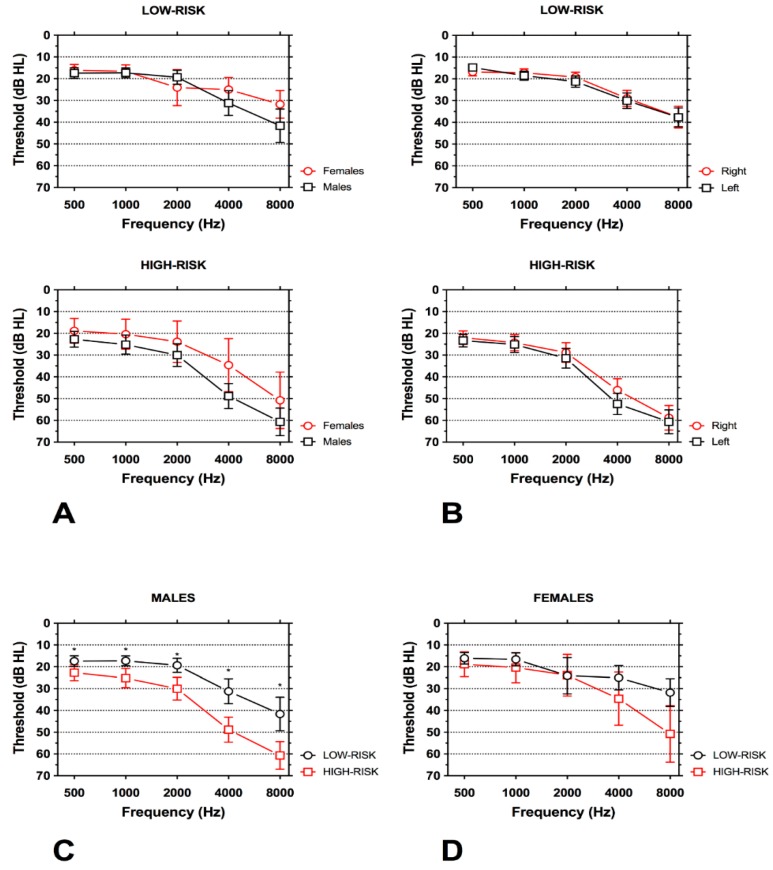
Pure tone audiogram (PTA) in the LOW-RISK and HIGH-RISK groups showing differences between males and females and side. Means ±95 CI are shown. (**A**) Worse hearing thresholds were found in males; however, the difference within the same group was not significant (*p* = 0.086). (**B**) No significant differences were found in hearing threshold between the right and the left ear although thresholds for high frequencies in the left ear were worse compared to the right ear (*p* = 0.64). (**C**) PTA for males; individuals in the HIGH-RISK group had a significantly worse hearing threshold than individuals in the LOW-RISK group (*p* < 0.001). (**D**) PTA for females; although worse hearing for high frequencies was found in patients in the HIGH-RISK group, the difference was not statistically significant (*p* = 0.12).

Figure 2 shows the average PTA for males and females and right and left ear in both groups. No statistically-significant differences between gender (*p* = 0.086) and side (*p* = 0.64) were found within the same groups; however, the left ear showed poorer mean auditory thresholds for higher frequencies in the HIGH-RISK group compared to the right ear. Although worse hearing, especially for high frequencies, was found in the HIGH-RISK group compared to the LOW-RISK group for both males and females, a larger and statistically-significant difference was found for males (*p* < 0.001), not for females (*p* = 0.12).

### 3.3. Tinnitus Characteristics and Self-Administered Questionnaires Scores

Average duration of tinnitus at the time of first admission to our center was 10.9 years for the HIGH-RISK group and 9.2 years in the LOW-RISK group. The difference was not statistically significant (*p* = 0.726). Tinnitus was bilateral in 46/68 (67.6%) patients in the HIGH-RISK group and in 36/68 (52.9%) in the LOW-RISK group (*p* = 0.05). Unilateral tinnitus was significantly more prevalent in the left ear; left-sided tinnitus was found in 18/22 (81.8%) individuals in the HIGH-RISK group and in 19/32 (59.3%) in the LOW-RISK group (*p* = 0.05). Tinnitus was described as “whistle” in 46/136 (33.8%) patients, “buzzing” in 30/136 (22.1%), “high-pitched” in 26/136 (19.1%), “low-pitched” in 15/136 (11%) and “other” in 19/136 (13.9%) (*p* = 0.06). “Buzzing” and “high-pitched” tinnitus sounds were more common among HIGH-RISK individuals, and “whistle” was more common among patients in the LOW-RISK group.

Mean THI score was 33.1 in the HIGH-RISK group and 30.6 in the LOW-RISK group; the mean HHI score was 18.8 in the HIGH-RISK group and 9.4 in the LOW-RISK group; the HQ score was 13.4 in the HIGH-RISK group versus 11.8 in the LOW-RISK group. The difference was not significant for THI (*p* = 0.22) and HQ (*p* = 0.12); a statistically-significant difference was found for HHI (*p* < 0.001). Table 3 shows detailed data for tinnitus characteristics and questionnaire scores for the HIGH-RISK and LOW-RISK groups.

### 3.4. Differences among Occupations

Differences in demographics, tinnitus onset and laterality, self-administered questionnaire responses and hearing loss were found in relation to the different occupations reported by patients. 

In the HIGH-RISK group, female gender was more prevalent among manufacturing workers and school teachers, while the male gender prevailed among all other occupations. Tinnitus was mostly bilateral in school teachers (91.6%), miners (75%), construction workers (73.3%) and armed forces (72.7%); unilateral in railroaders (66.6%) and musicians (100%). The worst THI scores were found for school teachers (50.5) and best among musicians (21) and armed forces (24.1). Manufacturing workers (23.5) and construction workers (23.4) scored worst for HHI. Surprisingly, railroaders had the best HHI score (2.6). Worst hearing thresholds were found in miners (47.5 dB for 0.5–2 kHz and 78.1 dB for 4–8 kHz) and railroaders (31.6 dB for 0.5–2 kHz and 65.8 dB for 4–8 kHz). Musicians had the best hearing threshold among individuals in the HIGH-RISK group (11.6 dB for 0.5–2 kHz and 33.7 dB for 4–8 kHz). 

In the LOW-RISK group, bilateral tinnitus was more prevalent among entrepreneurs (63.6%) and office workers (54.2%) and unilateral among hospital workers (75%). The worst THI score was found among office workers (33.7); the worst HHI score among entrepreneurs (13.18). The worst hearing thresholds were found for professionals (23.2 dB for 0.5–2 kHz and 40.9 dB for 4–8 kHz); hospital workers had the best hearing among individuals in the LOW-RISK group (13.3 dB for 0.5–2 kHz and 15 dB for 4–8 kHz). Data sorted by type of work are shown in Table 4.

### 3.5. The Role of Age in Relation to Tinnitus, Hearing Characteristics and Questionnaire Scores

**Figure 3:**
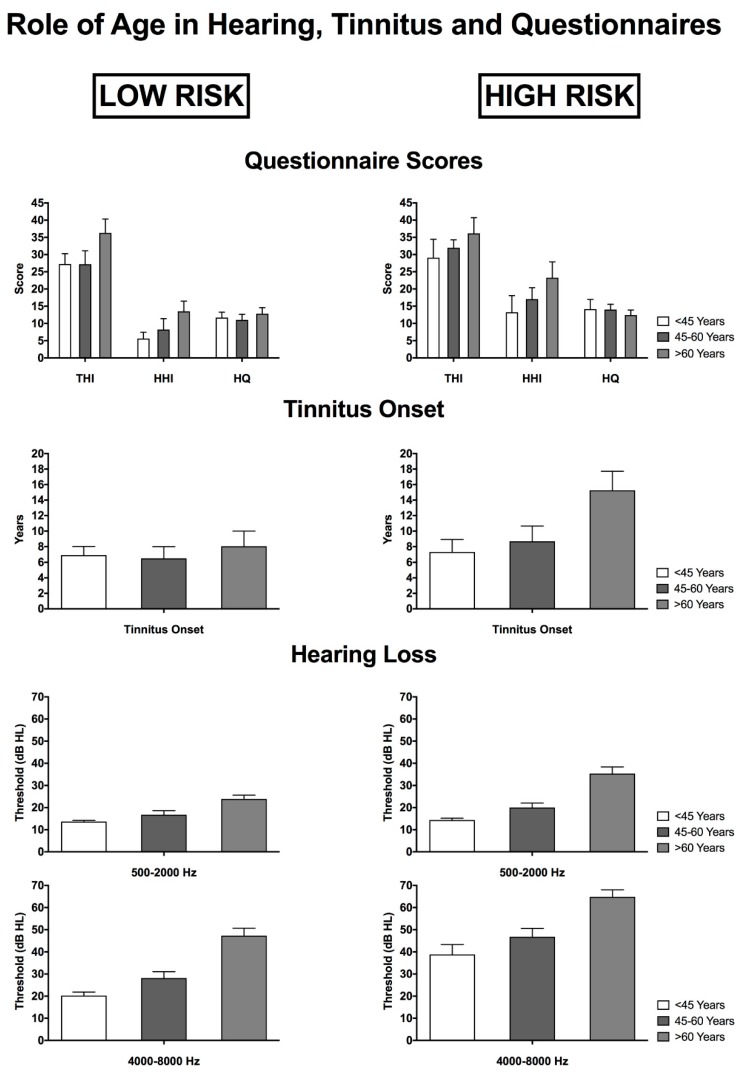
Relationship between age of the patient and hearing loss (PTA), tinnitus onset and self-administered questionnaire scores (HHI, THI, HQ) sorted by LOW-RISK and HIGH-RISK groups.

The role of age in relation to tinnitus onset, hearing threshold and THI, HHI and HQ scores was evaluated for both groups. In the LOW-RISK group, younger patients (<45 years) showed significantly lower THI and HHI scores (*p* = 0.001) and PTA for the 0.5–2-kHz (*p* = 0.05) and the 4–8-kHz frequency range (*p* < 0.001) compared to older subjects (>60 years). No significant differences were found for HQ score and tinnitus length. In the HIGH-RISK group, compared to participants older than 60 years, patients younger than 45 years showed a significantly lower length of tinnitus (*p* = 0.02), PTA for the 0.5–2-kHz (*p* < 0.001) and the 4–8-kHz frequency range (*p* < 0.001). No significant differences were found for THI, HHI and HQ scores (Figure 3). 

**Figure 4:**
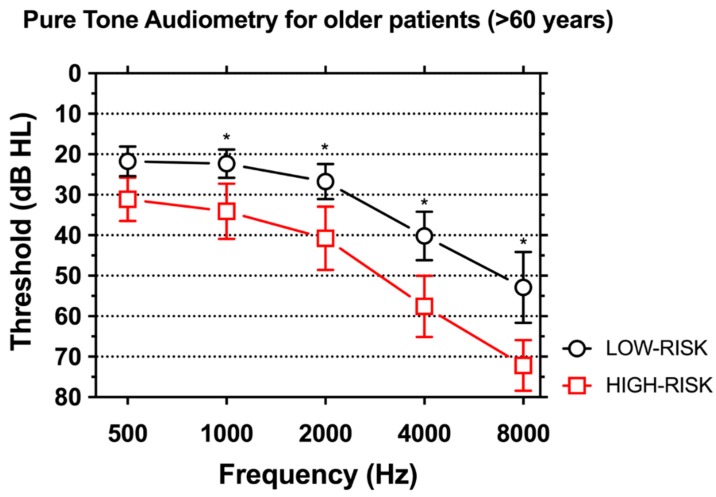
Comparison of pure tone audiometry thresholds in subjects older than 60 years in the LOW-RISK and HIGH-RISK groups. Significantly worse hearing was found in individuals in the HIGH-RISK group for all frequencies above 500 Hz (*p* < 0.001). Asterisks indicate statistically-significant differences.

When analyzing hearing loss for single frequencies, older (>60 years) individuals showed significantly worse hearing in the HIGH-RISK group compared to the LOW-RISK group for all frequencies above 500 Hz (*p* < 0.001) (Figure 4).

A multivariate binary logistic regression analysis was used to investigate specific variables associated with a higher degree of hearing loss in tinnitus patients according to demographic characteristics such as age and sex, comorbidities, family history for hearing loss and the HHI self-administered questionnaire score. Analysis indicated that patients with a higher degree of hearing loss: (A) were 3.54-times more probable to come from male populations; (B) were 1.7-times more likely to have a family history of hearing loss; and (C) were 1.2-times more likely to have at least one comorbidity (Table 5).

## 4. Discussion

The association between hearing loss, tinnitus and occupation has been previously demonstrated [34,43,65,66,67,68,69,70,71]. The aim of this study was to survey patients with chronic tinnitus with and without a history of long-term work-related noise exposure, comparing demographic variables, tinnitus and hearing loss characteristics and self-administered questionnaire responses for tinnitus, hearing loss and hyperacusis. Significant differences were found between groups for gender, auditory threshold and tinnitus laterality. Individuals employed in jobs with a high risk of noise exposure were mostly males and had a poorer hearing threshold, more evident in the left ear, although the difference with the right ear was not significant; tinnitus was mostly bilateral, followed by left-sided, described as buzzing or high-pitched. Correlation between age, length of tinnitus and worse hearing was found. Patients with a higher degree of hearing loss were mostly males and were likelier to have a family history of hearing loss and at least one cardiovascular comorbidity.

### 4.1. Main Differences for Gender, Age, Family History and Comorbidities

The main demographic difference found among our groups was for the male gender. The larger prevalence of males found between individuals in the HIGH-RISK group compared to the LOW-RISK group (80.8% vs. 45.6%) is in accordance with other studies that show that men are mostly involved in jobs with elevated noise exposure [68,72,73]. Within different professions, females were more prevalent among school teachers and manufacturing workers in the HIGH-RISK group and among hospital workers and professionals in the LOW-RISK group.

Mean age did not differ between groups; however, a significant difference was found between patients younger than 45 years and older than 60 years for auditory thresholds and length of tinnitus. Older individuals had worse hearing thresholds and experienced tinnitus for a longer time. This is consistent with the literature, which reports greater incidence of tinnitus and hearing loss with age [24,25,26,68,69,74]. When comparing older (>60-year-old) individuals in the two groups, significantly worse hearing was found in patients in the HIGH-RISK group, suggesting that such a trend is accelerated in patients exposed to noise in general and, more specifically, to noisy working environments [68,69].

Although the degree of NIHL has been shown to be significantly influenced by environmental factors, strong evidence has been gathered through various animal and human studies about the role of genetic predisposition [75,76,77]. In our study, family history for hearing loss did not seem to be statistically different between groups. However, a larger percentage of patients in the HIGH-RISK group reported a positive history (20.6%) compared to the LOW-RISK group (13.2%). Furthermore, by binary logistic regression analysis, patients with a higher degree of hearing loss were 1.7-times more likely to have a family history of hearing loss.

The presence of cardiovascular comorbidities in individuals with NIHL has been previously described [78,79,80,81]. In our sample, 27/68 (39.7%) patients in the HIGH-RISK group had at least one comorbidity, predominantly hypertension and vascular diseases. Although we could not find a statistical difference with patients in the LOW-RISK group, our findings are in accordance with the literature that shows a well-established relationship between hearing loss, diabetes and heart disease [82]. Diabetes represents a risk factor for early-onset NIHL, as high blood sugar may cause a reduction in the caliber of blood vessels in the inner ear and especially in the stria vascularis [83,84,85]. Similarly, cardiovascular diseases have been shown to increase the risk of hearing loss [86]. In addition, exposure to loud noise has been shown to have non-auditory long-term effects that may include elevated blood pressure, loss of sleep and increased heart rate [82,87]. 

### 4.2. Characteristics of Hearing Loss in Subjects at High-and Low-Risk for Work-Related Hearing Loss

Among individuals with chronic tinnitus, hearing thresholds were significantly worse in patients in the HIGH-RISK group compared to those in the LOW-RISK group. This finding is in accordance with the literature [3,4,5,34,35,36,37,38,39,41,42,43,44,45,47,48,49,50,51,54,55,56,57,58,68,70,74,88]. Our results showed a worse, although not significant, hearing threshold for high frequencies in the left ear compared to the right among individuals in the HIGH-RISK group; no side difference was found in the LOW-RISK group. Occupational noise was demonstrated to induce asymmetric hearing loss with higher impact on the left side compared to the right [70,88], with an incidence between 4.7% and 36% [70]. Asymmetries are usually inferior to 5 dB and tend to increase at higher frequencies [89]. Such higher vulnerability of the left ear could be attributed to ambient exogenous noise-exposure factors, such as the “handedness” of the noise source for different occupations [70], or by endogenous factors, such as neuroanatomic differences between the left and right parts of the auditory system, with involvement of the protective role of the efferent pathways to cochlea [69]. Tinnitus was also reported to be more frequent in the left ear than the right ear [70,72]. 

One possible explanation for this phenomenon is the different shielding of the right ear from noise in specific occupations. An example of a work environment resulting in asymmetrical noise exposure is tractor drivers, in which the left ear is more frequently affected than the right ear, as these operators monitor equipment mounted on the rear side looking over their right shoulder and therefore exposing their left ear to the noise while their right ear is shielded by head shadow. The acoustic shielding of the head is also usually found in right-handed shooters that have a more severe hearing loss in the left ear. The handedness of the subject could thus be of relevance; however, studies assessing the impact of handedness on hearing loss showed no correlation between the ear with the asymmetry and the individual’s handedness [88]. To date, the reasons for asymmetric hearing loss following noise exposure are still unclear and need further research. 

### 4.3. Tinnitus Characteristics: Laterality, Pitch, Annoyance

The main difference in tinnitus characteristics among individuals in the HIGH-RISK and LOW-RISK groups was laterality. A significantly higher number of individuals in the HIGH-RISK group had bilateral tinnitus. Among patients with unilateral tinnitus, a strong prevalence of left ear tinnitus was found in patients in the HIGH-RISK group (81.8% vs. 59.3%). Our findings are in accordance with other studies [32,68,69,70,88] and consistent with the auditory asymmetry generally documented in NIHL [69,70,72,88,89] and in our study.

Consistent with findings in a recent paper by Flores [68], no association between pitch of tinnitus and frequency of hearing loss could be found in our sample. However, our results are in disagreement with those by Schecklmann, who analyzed the relationship between audiometric slope and tinnitus pitch in 286 patients and reported that the pitch of tinnitus was associated with the frequency of the greatest hearing loss [73]. Our relatively small cohort could explain the missed statistical significance for our data.

No significant differences were found for mean THI questionnaire scores between our groups, in contrast to other authors who showed a higher tinnitus discomfort in individuals with NIHL [69,90]. When looking at THI in specific working categories, a direct relationship with the hearing threshold was found for miners and railroaders, two categories in which patients reported poor hearing thresholds and relatively elevated THI scores. However, the worst THI scores were found among manufacturing workers, a category of workers that showed limited hearing loss in our study. This may be due to non-auditory elements, such as the psychological factors, that affect the self-perception of the disorders. Higher tinnitus loudness, discomfort and annoyance in this category could be therefore explained by the involvement of emotion-related neural circuits [91,92]. 

### 4.4. Study Limitations

This is one of the few studies on work-related noise exposure to include only individuals with chronic tinnitus and a long working history. Accurate audiological and tinnitus evaluation was uniformly performed among groups, although it was limited to PTA and did not investigate outer hair cell functions with otoacoustic emissions. Acuphenometry for pitch and loudness of tinnitus was not performed; pitch was investigated through an anamnestic interview; psychometric scores were used to assess the degree of tinnitus severity instead of investigating its psychoacoustic characteristics. Studies report that mood disorder comorbidity among individuals with tinnitus can be as high as 60–80% and can lead to increases in measures of tinnitus annoyance [93,94]. Therefore, extra-auditory characteristics must be considered when evaluating tinnitus annoyance and its relationship to hearing loss. 

A limitation of this study is the lack of information about the loudness of noise exposure and about the degree to which workplace prophylaxis might have been used to mitigate the work-related hazard for individuals included in the study. However, assignment to the HIGH-RISK or LOW-RISK groups was done according to extensive evidence reported in large demographical studies [5,34,38,43] and recommended by the U.S. National Institute for Occupational Safety and Health (NIOSH).

Hearing loss in the range of 10–16 kHz was not investigated in the present study. Such high-frequency hearing loss can be found in many individuals above the age of 40 and is common in noise-exposed subjects [8,9]. Hearing loss above the clinical range has been studied with high-frequency audiometry in occupational noise-exposed individuals. High-frequency hearing loss has been suggested as an early indicator of NIHL, and high-frequency audiometry has been proposed for assessing susceptibility to noise damage [95,96,97].

The relatively small size of our study cohort did not allow a uniform distribution of individuals among the different job categories. A large heterogeneity of noise exposure levels and timing of exposure can be found in our sample and may have biased results. A larger sample size may have improved the significance of our data and allowed us to examine a larger number of occupations. Furthermore, although no significant differences for length of noise exposure between groups were found, correlation between time of occupational noise exposure and audiological and tinnitus characteristics in exposed subjects was not performed in our sample and could be further explored in future studies.

No historical audiological data were collected for patients, preventing us from differentiating hearing losses due to noise exposure, ototoxic agents or a combination of exposures and, therefore, to correlate the degree of hearing loss found in our study exclusively with work-related noise exposure. 

## 5. Conclusions

Our study shows some differences in individuals with tinnitus and a history of a profession associated with an increased exposure to occupationally-acquired noise-induced hearing loss compared to those who had no such history. Individuals employed in jobs at high risk for NIHL were mostly males and had a poorer hearing threshold, more evident in the left ear; tinnitus was mostly bilateral, followed by left-sided, described as buzzing or high-pitched. Correlation between age, length of tinnitus and worse hearing was found. Patients with a higher degree of hearing loss were mostly males and were more likely to have a family history of hearing loss and at least one cardiovascular comorbidity.

## Figures and Tables

**Table 1:** Distribution of demographic characteristics between individuals with tinnitus in the LOW-RISK and HIGH-RISK groups. A significant prevalence of male gender was found in the HIGH-RISK group. No significant differences were found for age, time of noise exposure, family history of noise exposure and cardiovascular comorbidities between the two groups.

Demographic Characteristics	LOW-RISK	HIGH-RISK	*p*-Value
*Age (mean (SD))*	53.5 (13.5)	56.6 (12.4)	0.08
Male (freq. (%))	37 (54.4)	55 (80.9)	0.001
Female (freq. (%))	31 (45.6)	13 (19.2)	0.001
*Family history (freq. (%))*			
No hearing loss	59 (86.8)	54 (79.4)	
Hearing loss	9 (13.2)	14 (20.6)	0.253
Time of noise exposure in years (mean (SD))	18.4 (8.1)	19.3 (6.7)	0.72
*Comorbidity (freq. (%))*			
No comorbidity	44 (64.7)	41 (60.3)	
At least one comorbidity	24 (35.3)	27 (39.7)	
Heart disease	7 (29.2)	5 (18.5)	
Diabetes	4 (16.7)	3 (11.1)	
Hypertension	18 (75)	21 (77.8)	
Vascular diseases	4 (16.7)	6 (22.2)	0.60

**Table 2:** Pure tone audiometry (PTA) analysis in the LOW-RISK and HIGH-RISK groups. Significant differences between groups were found for all frequencies for average, right and left ear thresholds.

PTA	LOW-RISK	HIGH-RISK	*p*-Value
	*Average Right/Left Ear (mean (SD))*		
500 Hz	16.8 (7.2)	22.0 (12.6)	0.002
1000 Hz	17.0 (7.2)	24.3 (15.3)	<0.001
2000 Hz	21.5 (16.8)	28.8 (18.4)	0.008
4000 Hz	28.4 (16.3)	46.1 (21.4)	<0.001
8000 Hz	37.1 (20.9)	58.8 (23.2)	<0.001
	*Right Ear (mean (SD))*		
500 Hz	16.8 (7.2)	22.0 (12.6)	0.004
1000 Hz	17.2 (7.2)	24.3 (15.3)	<0.001
2000 Hz	19.2 (9.2)	28.8 (18.4)	<0.001
4000 Hz	29.0 (15.1)	46.1 (21.4)	<0.001
8000 Hz	37.6 (20.3)	58.8 (23.1)	<0.001
	*Left Ear (mean (SD))*		
500 Hz	16.8 (7.2)	23.3 (11.8)	<0.001
1000 Hz	17.0 (7.2)	25.1 (14.9)	<0.001
2000 Hz	18.8 (9.5)	31.5 (18.4)	<0.001
4000 Hz	29.0 (15.9)	52.4 (19.9)	<0.001
8000 Hz	38.2 (20.3)	60.7 (22.3)	<0.001

**Table 3:** Distribution of tinnitus characteristics and questionnaire scores in the LOW-RISK and HIGH-RISK groups. A significantly higher number of patients in the HIGH-RISK group had bilateral tinnitus, followed by unilateral tinnitus in the left ear. “Buzzing” and “high-pitched” tinnitus sounds were more common among HIGH-RISK individuals; “whistle” was more common among individuals in the LOW-RISK group. Patients in the HIGH-RISK group scored significantly worse for the Hearing Handicap Inventory (HHI) questionnaire compared to individuals in the LOW-RISK group; no significant differences were seen for the Tinnitus Handicap Inventory (THI) and the Hyperacusis Questionnaire (HQ).

Tinnitus Characteristics and Questionnaire Scores	LOW-RISK	HIGH-RISK	*p*-Value
*Tinnitus side (freq. (%))*			
Left	19 (27.9)	18 (26.5)	0.05
Right	13 (19.1)	4 (5.9)	
Bilateral	36 (52.9)	46 (67.6)	
*Tinnitus Sound (freq. (%))*			
Buzzing	11 (16.2)	19 (27.9)	0.06
High-pitched	9 (13.2)	17 (25.0)	
Low-pitched	7 (10.3)	8 (11.8)	
Other	12 (17.6)	7 (10.3)	
Whistle	29 (42.6)	17 (25.0)	
*Questionnaire scores (mean (SD))*			
THI	30.6 (18.1)	33.1 (18.8)	0.22
HHI	9.4 (13.4)	18.8 (20.3)	<0.001
HQ	11.8 (7.9)	13.4 (8.3)	0.12

**Table 4:** Demographics, tinnitus characteristics, questionnaire scores, and hearing loss metrics among job types. A, upper part of the table: jobs of patients in the HIGH-RISK group; B, lower part of the table: jobs of individuals in the LOW-RISK group.

Occupation	Male (%)	Age (y)	Work (y)	Bilateral Tin (%)	Tin onset (y)	THI	HHI	HQ	PTA (0.5–2 kHz)	PTA (4–8 kHz)
*HIGH-RISK*										
Armed Forces (*n* = 11)	100	54.8	19.9	72.7	9.8	24.1	11	9.7	16.8	44.5
Carpenters (*n* = 8)	100	54.2	14.7	62.5	9.7	29.7	21.7	15.1	24.7	52
Manufacturing Workers (*n* = 4)	0	44.5	11.2	50	8	50.5	23.5	16.2	25.4	46.2
Drivers (*n* = 9)	100	61.1	16.5	55.5	12.6	29.1	17.1	10.6	31.2	60
Miners (*n* = 4)	100	55	20.7	75	8.5	38	47	14.2	47.5	78.1
Musicians (*n* = 2)	100	47.5	13	0	6.5	21	30	22	11.6	33.7
Railroaders (*n* = 3)	100	61.3	21	33.3	15.3	42	2.6	8	31.6	65.8
School Teachers (*n* = 12)	33.3	63.7	21	91.6	16.6	33.6	15.1	17.4	22.3	45.6
Construction Workers (*n* = 15)	93.3	54.8	23	73.3	8.8	37	23.4	12.6	23.8	54.5
*LOW-RISK*										
Entrepreneurs (*n* = 11)	81.8	48.7	18.5	63.6	11.6	28	13.1	13.8	16.1	31.8
Hospital Workers (*n* = 4)	50	38.7	16.7	25	6.2	21	1.5	10	13.3	15
Office Workers (*n* = 35)	51.4	53.7	19.2	54.2	6.6	33.7	8.9	11.1	17.2	31.4
Professionals (*n* = 18)	44.4	59.5	21.8	33.7	9.5	27.8	11.9	9.6	23.2	40.9

y: year.

**Table 5:** Binary logistic regression analysis for variables such as age and sex, comorbidities, family history for hearing loss and HHI questionnaire score in patients with a higher degree of hearing loss. Statistically-significant results are shown in bold.

Variable	Odds Ratio	Confidence Interval	*p*-Value
Age	1.02	0.99–1.05	0.16
Male	3.54	1.64–7.66	0.001
Family history	1.70	0.68–4.24	0.26
Comorbidity	1.20	0.6–2.42	0.60
HHI	1.03	1.01–1.06	0.003
